# Rituximab-Induced Serum Sickness in Patients With Multiple Sclerosis: A Case Report and Literature Review

**DOI:** 10.7759/cureus.101911

**Published:** 2026-01-20

**Authors:** Asmae Sikkal, Salma Ouahmane, Hicham El Otmani, Bouchra El Moutawakil, Mohammed Abdoh Rafai

**Affiliations:** 1 Department of Neurology and Clinical Neurophysiological Explorations, Ibn Rochd University Hospital of Casablanca, Casablanca, MAR; 2 Laboratory of Genetics and Molecular Pathology, Faculty of Medicine and Pharmacy of Casablanca, Hassan II University, Casablanca, MAR; 3 Research Laboratory on Diseases of the Nervous System, Neurosensory and Handicap, Faculty of Medicine and Pharmacy of Casablanca, Hassan II University, Casablanca, MAR

**Keywords:** immune complex-mediated hypersensitivity reaction, multiple sclerosis, rituximab, rituximab-induced serum sickness, serum sickness

## Abstract

Rituximab is a chimeric anti-CD20 monoclonal antibody that is becoming increasingly used in multiple sclerosis. In rare cases, rituximab may induce a delayed immune-mediated reaction such as rituximab-induced serum sickness. We report the case of a 49-year-old woman with multiple sclerosis who developed rituximab-induced serum sickness characterized by fever, diffuse rash, lymphadenopathy, and arthralgia nine days after her first rituximab infusion. We reviewed the number of cases in a literature review, discussing their characteristics and mechanisms and the implications for using alternative anti-CD20 monoclonal antibodies such as ocrelizumab or ofatumumab for the management of multiple sclerosis.

## Introduction

Rituximab is a chimeric monoclonal IgG1 antibody that targets CD20-positive B lymphocytes, leading to their rapid depletion [1]. During the last few decades, rituximab has been widely used in the treatment of hematologic and autoimmune diseases. Although its use in multiple sclerosis remains off-label, rituximab has shown high efficacy in reducing inflammatory activity.

Rituximab is commonly associated with infusion-related reactions occurring during or shortly after administration, including fever, chills, and rigors, as well as allergic or anaphylactoid manifestations such as urticaria, angioedema, and hypotension [2]. These symptoms are caused by cytokine release in response to the murine portion of the antibody or B-cell lysis. Premedication with corticosteroids and antihistamines can substantially reduce the incidence and severity of these reactions [2].

In contrast, delayed immunologic reactions such as rituximab-induced serum sickness (RISS) are extremely rare [3]. RISS represents a type III hypersensitivity reaction, a mechanism mediated by the formation of circulating immune complexes composed of the drug and anti-drug antibodies. Consequently, symptom onset is delayed, typically occurring 7-21 days after exposure, and classically manifests with fever, rash, and arthralgia [2].

In this context, we present a case of RISS in a patient with multiple sclerosis to highlight the importance of early recognition and distinguish this syndrome from acute infusion reactions.

## Case presentation

A 49-year-old woman with relapsing-remitting multiple sclerosis was previously treated with azathioprine and interferon-beta. She was switched to rituximab because an MRI revealed new lesions, including a contrast-enhancing cerebral lesion. The first 1000 mg rituximab infusion was well tolerated, with no immediate infusion-related reactions. Nine days later, she developed fever up to 39°C, profound fatigue, generalized tender lymphadenopathy, diffuse arthralgia, a generalized maculopapular rash, and trismus. No infectious etiology was identified. C-reactive protein (CRP) and erythrocyte sedimentation rate were high, with thrombocytopenia (63; normal 145-390×109/L), and complement levels were normal as shown in Table 1. Based on the temporal profile and triad of fever, arthralgia, and erythematous rash following drug exposure, a diagnosis of RISS was made. The levels of human anti-chimeric antibodies (HACA) were not measured. The patient was admitted to the intensive care unit and received a single intramuscular dose of prednisone (80 mg), followed by oral prednisone (1 mg/kg) and oral levocetirizine for five days. Fever and other symptoms rapidly resolved within the next 48 hours, and inflammatory markers normalized. 

**Table 1:** Laboratory findings at presentation

Laboratory parameter	Result	Reference range
C-reactive protein	60 mg/L	<5 mg/L
Erythrocyte sedimentation rate	100 mm/h	<20 mm/h
Platelet count	63×10⁹/L	145-390×10⁹/L
White blood cell count	6×10⁹/L	4-10×10⁹/L
Hemoglobin	13.5 g/dL	12-16 g/dL
Complement C3	Normal	0.9-1.8 g/L
Complement C4	Normal	0.1-0.4 g/L

## Discussion

Herein, we describe a rare case of a delayed immunologic reaction following rituximab infusion. RISS remains a clinical diagnosis, and all neurologists should be aware of this complication given the expanding use of rituximab and other anti-CD20 monoclonal antibodies in multiple sclerosis practice.

RISS is a type III hypersensitivity reaction caused by the tissue deposition of immune complexes following rituximab infusion. The underlying mechanisms for the development of this reaction have been explained by two main mechanisms: B-cell lysis releases intracellular antigens that form immune complexes with antibodies, and the murine component of rituximab triggers the production of HACA. This antigen-antibody complex gets deposited in tissues and activates the complement cascade, resulting in the consumption of C3 and C4 [4]. However, complement levels may remain normal in the early phase of the disease, and normal complement levels do not exclude the diagnosis of RISS, reinforcing its primarily clinical nature [5].

We reviewed the number of cases in which RISS was described in multiple sclerosis patients treated with rituximab by conducting a MEDLINE search for patients using the following descriptors: "Rituximab-induced serum sickness", "serum sickness", "immune complex disease", "rituximab", "multiple sclerosis", and "MS". Cases that included therapies other than rituximab were excluded. Within these articles, we found nine cases of RISS in patients with multiple sclerosis as shown in Table 2 [3,6].

**Table 2:** RISS in multiple sclerosis patients: characteristics, evaluation, treatment, and outcomes n.a.: not available, (+): positive, (-): negative; DMT: disease-modifying treatment; RTX: rituximab; RISS: rituximab-induced serum sickness; CRP: C-reactive protein; ESR: erythrocyte sedimentation rate; WBC: white blood cell; HACA: human anti-chimeric antibodies

Series		Sex/age	Prior DMT	RTX infusion (number/dose)	Day of RISS	Symptoms	CRP	ESR	WBC (×10⁹/L)	C3/C4	HACA	Treatment for RISS	Outcome	Subsequent therapy	Recurrent RISS
Wolf et al. [3]	(1)	F, 69	Glatiramer acetate, fingolimod, dimethyl fumarate	Second, 1000 mg	7	Fever, rash, arthralgia	High	High	n.a.	Normal	+	Oral prednisone 40 mg×5 days	Recovered	Glatiramer acetate	n.a.
	(2)	F, 50	Interferons, daclizumab, dimethyl fumarate, glatiramer acetate, fingolimod, natalizumab	Second, 1000 mg	11	Rash, arthralgia	n.a.	n.a.	n.a.	n.a.	+	None	Recovered	Ocrelizumab	No
	(3)	M, 31	Dimethyl fumarate	First, 1000 mg	14	Fever, rash, arthralgia	High	High	n.a.	n.a.	+	None	Recovered	Fingolimod	n.a.
	(4)	F, 27	None	First, 1000 mg	10	Fever, rash, arthralgia	n.a.	n.a.	n.a.	Normal	-	IV methylprednisolone 500 mg×3 days+oral prednisone×10 days	Recovered	Ofatumumab	No
	(5)	F, 31	Glatiramer acetate, fingolimod	First, 1000 mg	7	Fever, rash, arthralgia	Normal	Normal	n.a.	Normal	n.a.	IV methylprednisolone	Recovered	Natalizumab	n.a.
Holmøy et al. [6]	(1)	M, 43	Interferons	First, 1000 mg	10	Fever, arthralgia	High	High	9.2	n.a.	+	None	Recovered	Ocrelizumab	No
	(2)	F, 23	Interferons, dimethyl fumarate	First, 1000 mg	12	Fever, arthralgia	High	n.a.	11.1	n.a.	+	Non-steroidal anti-inflammatory drugs	Recovered	Ofatumumab	No
	(3)	F, 34	None	First, 1000 mg	10	Fever, arthralgia, headache	High	High	5.2	n.a.	+	Steroids	Recovered	Ocrelizumab	No
Our patient		F, 49	Azathioprine, interferons	First, 1000 mg	8	Fever, rash, arthralgia	High	n.a.	5.8	Normal	n.a.	IV hydrocortisone 5 days+oral steroids+antihistamines	Recovered	Ocrelizumab	n.a.

Although five of nine patients (55%) presented with the classic triad of fever, rash, and arthralgia, this combination is reported in only 40-50% of cases [2]. The majority developed RISS after the first infusion (7/9; 77%), while the remaining two developed RISS after a second infusion, typically 1-2 weeks after rituximab infusion. The laboratory findings were heterogeneous, highlighting that the diagnosis of RISS remains clinical. In fact, laboratory abnormalities may not be altered in the acute phase of RISS in some cases [5]. In some cases, laboratory abnormalities, including complement consumption or inflammatory markers, may be absent in the acute phase, making biological confirmation unreliable [6].

Among the patients tested for HACA, six of seven (85%) were positive; however, in a cross-sectional series, 37% of multiple sclerosis patients treated with rituximab had anti-drug antibodies although none of them presented RISS [7]. Thus, the presence of HACA is not sufficient for RISS; in a systematic review including 33 cases of RISS, 40% of the tested patients were negative for HACA [2]. To date, the factors that contribute to immune complex formation and downstream complement activation and immunogenicity remain unclear [7].

All patients who were administered ocrelizumab or ofatumumab tolerated retreatment. Indeed, after an episode of RISS, it would be acceptable not to repeat infusions of rituximab due to possible recurrent episodes and propose fully humanized monoclonal anti-CD20 antibodies, such as ofatumumab or ocrelizumab. Humanized antibodies are expected to be less immunogenic than chimeric rituximab [8]. Moreover, mutagenesis and epitope mapping studies have shown differences between the CD20 epitopes recognized by these antibodies [9]. While ocrelizumab and rituximab target nearly identical epitopes on CD20, ocrelizumab appears less likely to trigger serum sickness-like reactions [8]. In contrast, ofatumumab binds to an epitope on CD20 with no overlap with the epitope interacting with rituximab and ocrelizumab [10].

## Conclusions

RISS is an uncommon but potentially serious complication that must be distinguished from acute infusion reactions. Its diagnosis remains primarily clinical, and differentiating RISS from acute infusion reactions and infections may be challenging due to heterogeneous laboratory findings, including normal complement levels in some cases. Key elements supporting the diagnosis include delayed symptom onset, systemic manifestations, and a clear temporal relationship with rituximab exposure. Given the potential risk of recurrence, further rituximab infusions should be avoided, and fully humanized anti-CD20 monoclonal antibodies should be considered as a safe alternative. Future studies are needed to identify patients at increased risk, refine diagnostic criteria, and better define the role of biomarkers, including complement levels and anti-drug antibodies, as well as to further evaluate the safety of other anti-CD20 monoclonal antibodies in multiple sclerosis. 
